# Supplementary material for: Meta-Analysis and Gene Set Enrichment Relative to ER Status Reveal Elevated Activity of MYC and E2F in the “Basal” Breast Cancer Subgroup
Source: PLoS One. 2009 Mar 9;4(3):e4710. doi: 10.1371/journal.pone.0004710 (PMC2650420; doi:10.1371/journal.pone.0004710)
Supplement: Dataset S1 — GSEA results from first screen. The file contains a zipped archive of the HTML results of the second GSEA screen. Reports for enriched or depleted datasets may be accessed from the “index.html” link within the my_analysis.GseaPreranked.1219975950625.rpt.zip ZIP archive. Enrichment plots are available for each of the 50 most enriched and depleted gene sets. (13.32 MB ZIP) [file pone.0004710.s001.zip › my_analysis.GseaPreranked.1219975950625.rpt.zip!//ZHAN_MM_CD138_PR_VS_REST.html]

Details for gene set ZHAN\_MM\_CD138\_PR\_VS\_REST[GSEA]

|  || Dataset | zSco.ERneg.to.ERpos\_collapsed |
| Phenotype | NoPhenotypeAvailable |
| Upregulated in class | na\_pos |
| GeneSet | ZHAN\_MM\_CD138\_PR\_VS\_REST |
| Enrichment Score (ES) | 0.7417741 |
| Normalized Enrichment Score (NES) | 2.6063154 |
| Nominal p-value | 0.0 |
| FDR q-value | 0.0 |
| FWER p-Value | 0.0 |
Table: GSEA Results Summary

  

Fig 1: Enrichment plot: ZHAN\_MM\_CD138\_PR\_VS\_REST      
 Profile of the Running ES Score & Positions of GeneSet Members on the Rank Ordered List

  

| PROBE | GENE SYMBOL | GENE\_TITLE | RANK IN GENE LIST | RANK METRIC SCORE | RUNNING ES | CORE ENRICHMENT || 1 | TTK |  |  | 11 | 7.059 | 0.0461 | Yes |
| 2 | FOXM1 |  |  | 21 | 6.760 | 0.0904 | Yes |
| 3 | TRIP13 |  |  | 96 | 5.890 | 0.1240 | Yes |
| 4 | MCM10 |  |  | 201 | 5.315 | 0.1515 | Yes |
| 5 | KIF14 |  |  | 221 | 5.215 | 0.1848 | Yes |
| 6 | BIRC5 |  |  | 229 | 5.191 | 0.2188 | Yes |
| 7 | TPX2 |  |  | 231 | 5.180 | 0.2531 | Yes |
| 8 | TSR1 |  |  | 241 | 5.138 | 0.2866 | Yes |
| 9 | BUB1B |  |  | 271 | 5.016 | 0.3178 | Yes |
| 10 | EZH2 |  |  | 312 | 4.888 | 0.3473 | Yes |
| 11 | ASPM |  |  | 359 | 4.767 | 0.3756 | Yes |
| 12 | RFC4 |  |  | 370 | 4.741 | 0.4063 | Yes |
| 13 | CCNB2 |  |  | 431 | 4.589 | 0.4323 | Yes |
| 14 | AURKA |  |  | 460 | 4.483 | 0.4600 | Yes |
| 15 | DLG7 |  |  | 521 | 4.306 | 0.4842 | Yes |
| 16 | PRC1 |  |  | 543 | 4.253 | 0.5109 | Yes |
| 17 | ZWILCH |  |  | 560 | 4.216 | 0.5377 | Yes |
| 18 | DCC1 |  |  | 567 | 4.206 | 0.5652 | Yes |
| 19 | SPBC25 |  |  | 606 | 4.126 | 0.5898 | Yes |
| 20 | TYMS |  |  | 829 | 3.738 | 0.5979 | Yes |
| 21 | NUSAP1 |  |  | 836 | 3.732 | 0.6223 | Yes |
| 22 | C18ORF24 |  |  | 894 | 3.635 | 0.6422 | Yes |
| 23 | UBE2C |  |  | 994 | 3.489 | 0.6580 | Yes |
| 24 | E2F8 |  |  | 1122 | 3.334 | 0.6706 | Yes |
| 25 | CDC2 |  |  | 1259 | 3.182 | 0.6815 | Yes |
| 26 | MAGEA6 |  |  | 1292 | 3.146 | 0.7000 | Yes |
| 27 | CCNB1 |  |  | 1626 | 2.823 | 0.6937 | Yes |
| 28 | MAGEA3 |  |  | 1653 | 2.798 | 0.7104 | Yes |
| 29 | NEK2 |  |  | 1703 | 2.758 | 0.7250 | Yes |
| 30 | KIAA1794 |  |  | 1724 | 2.747 | 0.7418 | Yes |
| 31 | RRM2 |  |  | 2047 | 2.485 | 0.7341 | No |
| 32 | SLC19A1 |  |  | 2675 | 2.105 | 0.7008 | No |
| 33 | GAGE4 /// GAGE5 /// |  |  | 2799 | 2.040 | 0.7051 | No |
| 34 | KPNA2 /// LOC643995 |  |  | 2876 | 1.999 | 0.7127 | No |
| 35 | TOP2A |  |  | 3735 | 1.610 | 0.6588 | No |
| 36 | GAGE2 /// GAGE4 /// |  |  | 3753 | 1.604 | 0.6682 | No |
| 37 | RACGAP1 |  |  | 4097 | 1.475 | 0.6522 | No |
| 38 | GAGE1 /// GAGE2 /// |  |  | 4260 | 1.409 | 0.6493 | No |
| 39 | GAGE7 |  |  | 4737 | 1.240 | 0.6217 | No |
| 40 | KIAA0101 |  |  | 4770 | 1.230 | 0.6275 | No |
| 41 | MAGEA1 |  |  | 7166 | 0.429 | 0.4500 | No |
| 42 | BRP44 |  |  | 11476 | -2.008 | 0.1389 | No |
Table: GSEA details [plain text format]

  

Fig 2: ZHAN\_MM\_CD138\_PR\_VS\_REST: Random ES distribution      
 Gene set null distribution of ES for **ZHAN\_MM\_CD138\_PR\_VS\_REST**
